# Ovarian Cancer or Hydatidosis? A Case Report

**Published:** 2018

**Authors:** Anahita NOSRATI, Eissa SOLEYMANI, Lotfollah DAVOODI

**Affiliations:** 1. Hematopathology-Molecular and Cytogenetic Unit, Pathology Department, School of Medicine, Shiraz University of Medical Sciences, Shiraz, Iran; 2. Research Committee, Razi Educational Hospital, Mazandaran University of Medical Sciences, Sari, Iran; 3. Antimicrobial Resistance Research Center, Mazandaran University of Medical Sciences, Sari, Iran

**Keywords:** Hydatidosis, Ovarian, Female, Iran

## Abstract

*Echinococcus granulosus* has been described as the common etiology of hydatid cysts in many parts of the world. A 54-yr-old female with lower abdominal pain referred to Gynecology Ward of Sari Imam Khomeini Hospital, Iran in 2016. Sonography was carried out and cysts in ovaries and liver were observed. The cysts of liver seemed to be hydatidosis but physicians were suspected about ovarian cystic mass. Anti-*Echinococcus* antibodies (ELISA) screen was positive. The operation was done on her and treatment by albendazole started one week before surgery and continued after discharge from the hospital. Pathology confirmed hydatidosis in ovary, also patient follow-up was performed for three months by abdominal CT scan that showed peritoan full of many small hydatid cysts. Uncommon locations for constitution of hydatid cysts such as ovary and peritoan often make the diagnosis very difficult. Hydatidosis is considered in differential diagnosis of any cysts of the entire body, especially in endemic countries such as Iran.

## Introduction

*Echinococcus granulosus* has been described as the most common culprit of the hydatid cysts. Hydatidosis is very common in some parts of the world such as East Europe, South America, Middle East, East Africa, Australia and Mediterranean area (1). This infection is one of the most important zoonotic diseases that occur in different parts of Iran (2). The adult worm is harbored in the intestine of carnivores like dogs, deemed as definitive hosts, (3) and the eggs are passed in the stool of the infected carnivores. Herbivores such as sheep, goats, cattle and humans accidentally are the intermediate host infected by ingestion of these eggs with water and food. In intermediate host oncospheres are hatched from the eggs in the intestine; and after invasion to the blood vessels, they can transfer into approximately all part of the body (4).

It does not typically has any sign but the site and size of the masses are important to development of symptoms (5). The liver was the most common location of the hydatid cyst, followed by the lung, with occurrence rates approximately of 70% and 12%, respectively (6,7). However, it may be found in any part of the body such as the heart, brain, and bones (8). Hydatidosis in genital organs is very rare compared with the involvement of other organs. Pure infection of genital organ is very unusual (9). Cystic echinococcosis of the ovary was found accidentally during laparotomy the ovarian tumor (10).

In this study, we present a rare case of bilateral ovary hydatid cyst associated with liver involvement.

## Case presentation

**Fig. 1:**
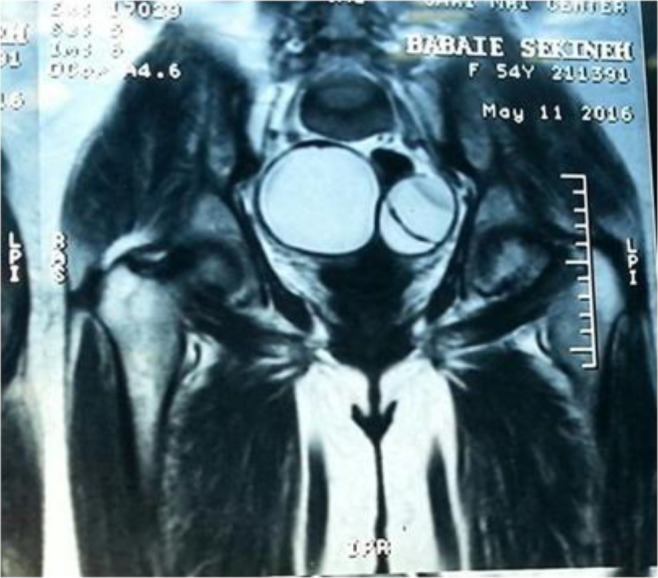
PELVIC MRI, coronal T2 view shows an 80*50 thin layer cyst in right ovarian and a multiloculated 75*40mm cyst in left ovary

A 54-year multipara and menopause woman with lower abdominal pain referred to Gynecology Ward of Imam Khomeini Educational Hospital Sari, Iran, in May 2016. She lived in rural region of Mazandaran Province, north of Iran. Clinical examination revealed a large smooth surfaced mass (10×10 cm) arising from pelvis with restricted mobility. Sonography of abdomen performed and cystic mass in both ovaries was seen. In addition, probably a cyst in liver hydatid was seen. MRI without contrast showed right ovarian simple cyst (80×50 mm) without internal septation and multiloculated left ovarian cyst (75×40 mm) was observed (Fig. 1).

All routine blood tests and tumors markers to be within the normal range such as CBC, biochemistry tests, CEA, CA125 and Alfa-Feto Protein performed were normal except ESR and LFT test that were abnormal. Anti-*Echinococcus* antibodies (ELISA) screen was positive. However ovarian cystic mass might be hydatidosis but to rule out malignancy, oophorectomy and hysterectomy were conducted.

**Fig. 2:**
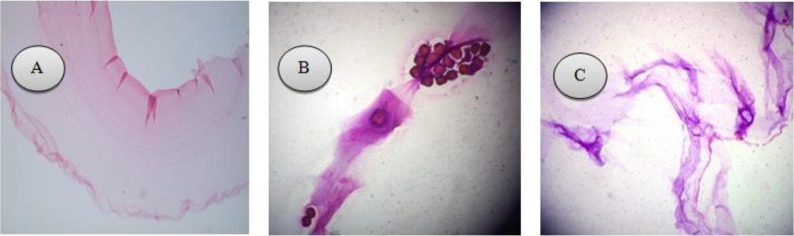
**A.** The endocyst consist of an internal cellular layer (germinal membrane). An outer layer composed of hyalinized, laminated, PAS-positive material. **B.** Hydatid cyst: some daughter cysts are evident. (*H&E* 10X) **C.** Hydatid cyst in ovary-unilocular cyst shows delicate fibrous laminar layer. (H&E 4X)

After bilateral oophorectomy, the cyst removed and sent to pathology laboratory. The pathology report confirmed hydatid cyst (Fig. 2).

**Fig. 3:**
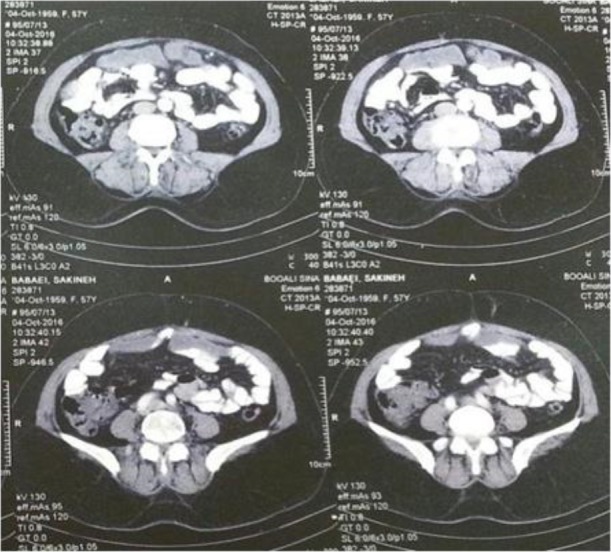
Spiral pelvic CT scan with IV&ORAL contrast shows multiple hypodense cysts in peritoen

Treatment with albendazole (400 mg) started one week before surgery and continued for three months. Postoperative follow-up was done for three months. CT scan performed for her indicated improvement of hydatidosis in liver but peritoan of this patient was full of many small hydatid cysts (Fig. 3). Albendazole for hydatidosis of peritoan continued.

Informed consent was taken from the patient and the study was approved by the Mazandaran University of Medical Sciences, Sari, Iran.

## Discussion

Generally, ovarian hydatidosis is very unusual (less than 1%). Typically this cyst presents a malignant tumor (11). In our case, clinical and paraclinical inspection confirm hydatidosis in liver, ovary, and peritoan of patient. In reviewing of the literature, we found less than ten papers about ovarian involvement in Iran. The disease was limited to ovary and fallopian tube (12).

Hydatid cyst in pelvic sites is a rare detection. The case of this paper presumably is a secondary hydatid cyst, due to rupture of liver cyst during surgery or spontaneously. Sterility, disorder of urinary tract system, distention and pain of abdomen and irregularity of menstruation are symptoms of pelvic hydatidosis (13). Ovarian echinococcosis can simulate either polycystic disease or malignancy (14, 15). Nonspecific symptoms and occasionally some deceptive imaging could contribute to misdiagnosis (14).

A high level of doubt or a preoperative diagnosis of *Echinococcus* cyst makes it possible to avoid an intraoperative iatrogenic rupture, and when available, to administer previously an Albendazole-based therapy in order to decrease the risk of dissemination that can lead to recurrences (16). Some ovarian tumor such as cystadenoma could have been spontaneously (17). Hydatid cysts expand slowly and asymptomatically, and thus, may be large at presentation (18). Pain is the most public symptom of hydatid disease, but this was absent in our case (19).

## Conclusion

Hydatidosis can exist in all part of the body and no site is immune. Uncommon locations such as ovary and peritoan frequently produce nonspecific signs; consequently, hydatid cyst is considered in various diagnoses of all cysts of the body, especially in endemic countries such as Iran.
